# Homology Modeling and Analysis of Structure Predictions of the Bovine Rhinitis B Virus RNA Dependent RNA Polymerase (RdRp)

**DOI:** 10.3390/ijms13078998

**Published:** 2012-07-19

**Authors:** Devendra K. Rai, Elizabeth Rieder

**Affiliations:** Foreign Animal Disease Research Unit, United States Department of Agriculture, Agricultural Research Service, Plum Island Animal Disease Center, Greenport, NY 11944, USA; E-Mail: devendra.rai@ars.usda.gov

**Keywords:** *Aphthovirus*, BRBV, homology modeling, 3D^pol^ structure predictions

## Abstract

Bovine Rhinitis B Virus (BRBV) is a picornavirus responsible for mild respiratory infection of cattle. It is probably the least characterized among the aphthoviruses. BRBV is the closest relative known to Foot and Mouth Disease virus (FMDV) with a ~43% identical polyprotein sequence and as much as 67% identical sequence for the RNA dependent RNA polymerase (RdRp), which is also known as 3D polymerase (3D^pol^). In the present study we carried out phylogenetic analysis, structure based sequence alignment and prediction of three-dimensional structure of BRBV 3D^pol^ using a combination of different computational tools. Model structures of BRBV 3D^pol^ were verified for their stereochemical quality and accuracy. The BRBV 3D^pol^ structure predicted by SWISS-MODEL exhibited highest scores in terms of stereochemical quality and accuracy, which were in the range of 2Å resolution crystal structures. The active site, nucleic acid binding site and overall structure were observed to be in agreement with the crystal structure of unliganded as well as template/primer (T/P), nucleotide tri-phosphate (NTP) and pyrophosphate (PPi) bound FMDV 3D^pol^ (PDB, 1U09 and 2E9Z). The closest proximity of BRBV and FMDV 3D^pol^ as compared to human rhinovirus type 16 (HRV-16) and rabbit hemorrhagic disease virus (RHDV) 3D^pols^ is also substantiated by phylogeny analysis and root-mean square deviation (RMSD) between C-α traces of the polymerase structures. The absence of positively charged α-helix at C terminal, significant differences in non-covalent interactions especially salt bridges and CH-pi interactions around T/P channel of BRBV 3D^pol^ compared to FMDV 3D^pol^, indicate that despite a very high homology to FMDV 3D^pol^, BRBV 3D^pol^ may adopt a different mechanism for handling its substrates and adapting to physiological requirements. Our findings will be valuable in the design of structure-function interventions and identification of molecular targets for drug design applicable to *Aphthovirus* RdRps.

## 1. Introduction

Viruses belonging to family *Picornaviridae*, cause a wide range of human and animal diseases. The family *Picornaviridae* consists of 12 distinct genera: *Enterovirus*, *Cardiovirus*, *Aphthovirus*, *Hepatovirus*, *Parechovirus*, *Erbovirus*, *Kobuvirus*, *Teschovirus*, *Sapelovirus*, *Senecavirus*, *Tremovirus* and *Avihepatovirus*. Picornaviruses are genetically diverse, small non-enveloped viruses with a single strand positive sense RNA genome of 7–8.9 Kb [1]. Their classification is based on physiochemical characteristics, genome and mode of replication.

*Rhinoviruses* were originally classified as separate genus of the family *Picornaviridae*. Based on available sequence and phylogenetic analysis, the International Committee on Taxonomy of Viruses (ICTV) has recently deleted the genus *Rhinovirus* and placed human rhinoviruses in the genus *Enterovirus* and bovine rhinoviruses in the genus *Aphthovirus* [2]. Bovine rhinitis viruses (BRV) are represented by two species, bovine rhinitis A virus (BRAV-1 and BRAV-2) and bovine rhinitis B virus (BRBV). The latter has only one serotype, bovine rhinitis B virus type-1 (BRBV-1). Initially a BRBV isolate EC-11, also known as BRV-2, was isolated from the lung of a specific pathogen-free calf that unexpectedly developed a respiratory disease [3]. In a recent study the virus was reported to be genetically closer to FMDV than HRV on the basis of phylogenetic characterization and full genome analysis [2].

The BRBV genomic RNA encodes a large polyprotein that is processed into structural (capsid) and non-structural polypeptide products by viral proteases during the infection. Picornavirus replication is catalyzed by the RdRp enzyme. RdRp is encoded by the 3D region of the genome at the *C*-terminus of the polyprotein and hence termed as 3D^pol^. The available crystal structures of a number of picornaviral 3D^pols^ show right hand structure with thumb, palm and finger domains common to all oligonucleotide polymerases [4–9]. 3D^pols^ have an additional fingertip region bridging finger and thumb domains [7].

In contrast to DNA polymerases that undergo conformational change to accommodate the substrate viral RdRps are not thought to involve conformational motions during the process of RNA replication. However, recent studies do show some evidence of conformational rearrangements in RdRps [10,11]. The crystal structure of FMDV RdRp in the unliganded form and in complex with nucleic acid and NTP revealed extensive similarities in terms of overall structural attributes with other RdRps [12]. The putative structural motifs involved in nucleotide recognition and binding (A and B), phosphoryl transfer (A and C), structural integrity of the palm domain (D) and priming nucleotide binding (E) [12,13] are conserved among polymerases [8,14–16]. Motif F in finger domain, a unique feature of RdRps, is involved in interactions with the incoming nucleotide [7].

The availability of the sequence information of the bovine rhinitis virus genome and the high resolution crystal structure of related RdRps (picornaviruses such as FMDV, poliovirus, coxsackievirus, HRV and caliciviruses such as RHDV) allow us to model the structure of the BRBV 3D^pol^ protein, which can potentially reveal new information about the general feature as well as specific properties of viral RdRps. Therefore, in this study we examined the functional domains, sequence features and the three dimensional structure of the modeled BRBV 3D^pol^, which could broaden our understanding of the structure-function relationship in RdRps among aphthoviruses.

## 2. Results and Discussion

### 2.1. Phylogenetic Analysis of BRBV RdRp

**Figure 1:**
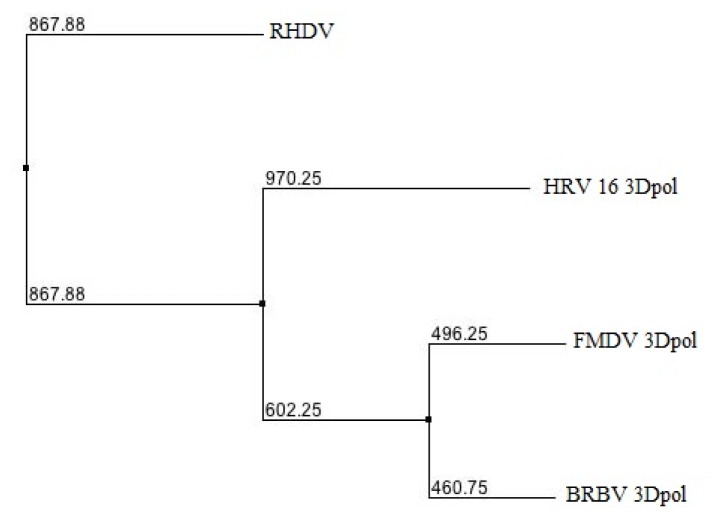
Phylogenetic analysis of Bovine Rhinitis B Virus (BRBV) 3D^pol^: An un-rooted tree showing the genetic relatedness of BRBV 3D^pol^ to other RdRps. The distance represents the difference from BRBV 3D^pol^ sequence conservation.

As shown in Figure 1, the distance map generated by BLOSUM62 scoring scoring matrix imbedded in Jalview platform [17,18] shows that BRBV 3D^pol^ is genetically closest to FMDV followed by HRV and RHDV 3D^pol^, respectively. The result supports the earlier study reporting close proximity of BRBV (previously known as BRV-2) to FMDV [3].

### 2.2. Structure Based Sequence Alignment

**Figure 2:**
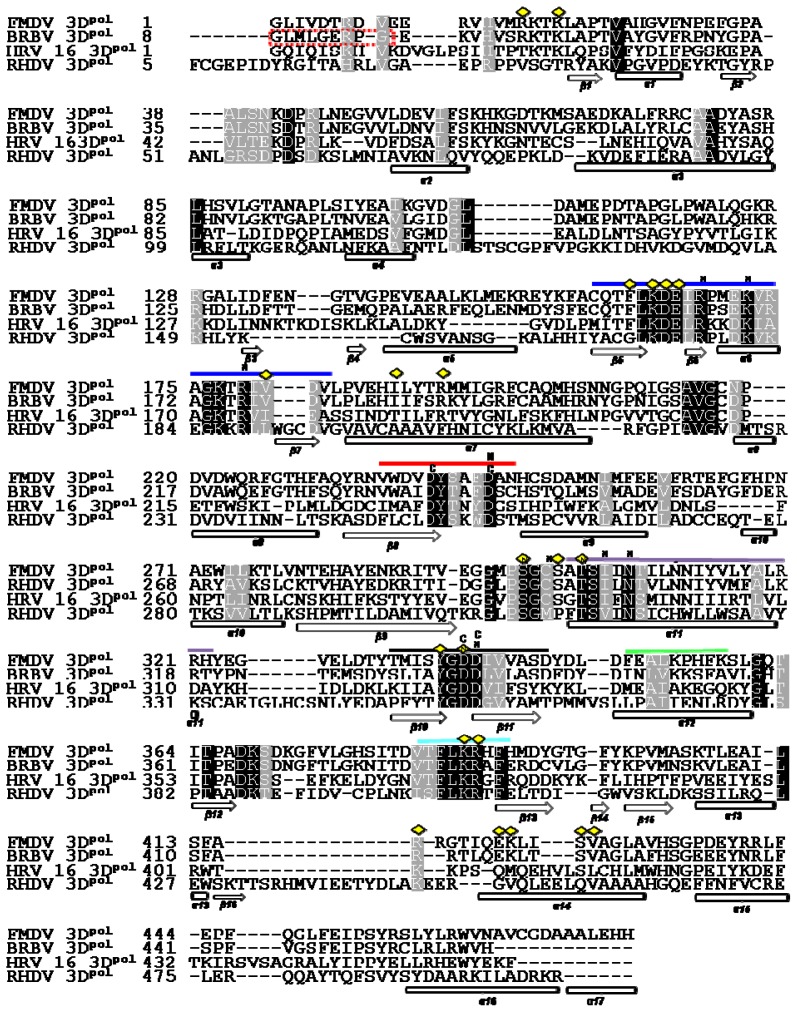
Structure based alignment of BRBV 3D^pol^ with other related RdRPs: The conserved regions are shaded black and gray to mark identical and similar residues, respectively. Each polymerase sequence is labeled starting from the left margin and the numbers next to polymerase in each row represent the sequence position. The red broken rectangle shows the sequence of BRBV 3D^pol^ excluded while preparing the homology model. Functionally important motifs A, B, C, D, E and F are marked with red, purple, black, green, light blue and blue bars, respectively. α-helices and β-sheets are numbered starting from the N-terminus of protein and marked with open cylinders and open arrows. Functional motifs A, B, C, D, E and F are marked with red, purple, black, green, light blue and blue bars, respectively. Their putative function has been discussed in the introduction. The nucleic acid binding residues are marked with the yellow diamonds. The nucleotide binding and catalytic sites are marked with the letters N and C. The motifs and other key structural features are based on the structure of FMDV 3D^pol^ described earlier [7].

The structure based alignment of the sequences of 3D^pol^ proteins of BRBV, FMDV, HRV-16 and RHDV was carried out using magic fit algorithm followed by iterative magic fit refinement on Deep View platform [19]. The alignment was verified and curated manually by looking at the superposed structures in order to define the secondary structure elements (SSEs) and functional motifs of all RdRps accurately. The results indicated that the BRBV 3D^pol^ shares 64.03% sequence identity with the FMDV protein counterpart, followed by HRV-16 3D^pol^ (29.39%) and RHDV 3D^pol^ (20.21%) respectively (Figure 2). BRBV 3D^pol^ shows 76.9, 94.12, 85.7, 45, 100 and 95.8% conservation for motifs A, B, C, D, E and F, respectively. These motifs are conserved in HRV-16 3D^pol^ as well, though to a lesser extent. BRBV, HRV-16 and FMDV 3D^pol^ seem to utilize the same T/P binding, NTP binding and active site. RHDV 3D^pol^ has a noticeably different T/P binding interface with fewer positive residues (Figure 2). The catalytic site is absolutely conserved among all the polymerases analyzed. The structure based sequence alignment suggests a close proximity between BRBV and FMDV 3D^pol^ followed by HRV-16 and RHDV 3D^pol^.

### 2.3. Search for Structural Homologues to BRBV 3D^pol^ via deconSTRUCT Web Server

The modeled structures of BRBV 3D^pol^ prepared by Geno3D and SWISS-MODEL [20–26] were submitted to the deconSTRUCT web server. dconSTRUCT finds homologous proteins via a totally different approach as discussed in the materials and methods section [27]. The details of the modeling protocol and analysis of structures are discussed in sections 2.4–2.8. The results from the structure based homologue search generated by deconSTRUCT suggested that the BRBV 3D^pol^ has the highest homology with the FMDV 3Dpol followed by Hepatitis C virus and RHDV polymerases (Table 1). However, as shown in Figure 2, when we superposed the structures of FMDV, BRBV, HRV-16 and RHDV RdRps and analyzed key structural motifs, BRBV 3D^pol^ was found to be closest to FMDV followed by HRV-16 and RHDV 3D^pol^. This discrepancy of prediction could be attributed to the methodology adopted by deconSTRUCT. Since it compares parts of proteins with fewer SSEs, this could result in a stronger signal for a distantly related protein. The superposition of structures on the other hand provides better control over comparison of overall as well as in part structure of proteins.

### 2.4. Preparation of Homology Model of BRBV 3D^pol^ and the Assessment of Model Accuracy

**Figure 3:**
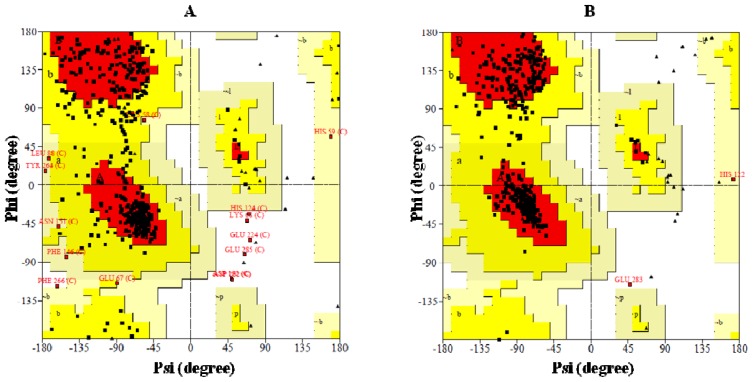
Ramachandran plot analysis of BRBV3D^pol^ structures: The Ramachandran plots of modeled BRBV structure generated by Geno3D (**A**) and SWISS-MODEL (**B**). The most favored regions are marked as {A, B, L}. The additional allowed regions are marked as {a, b, l, p}. All non-glycine and proline residues are shown as filled black squares, whereas, glycines (non-end) are shown as filled black triangles. Disallowed residues are colored red.

BRBV 3D^pol^ was modeled on FMDV and FMDV+HRV-16 3D^pol^ via SWISS-MODEL and Geno3D web servers, respectively. The stereochemical quality and accuracy of the models were tested using PROCHECK [28]. Results from PROCHECK are reported as Ramachandran plots (Figure 3). The main and side chain parameters are summarized in (Tables 2A and 3A) and (Tables 2B and 3B), respectively and discussed in the context of accuracy of the structure prediction. Ramachandran plot is one of the oldest yet most reliable methods of determination of the quality of protein structure. A structure with ≥90% of its residues in the most favored regions A, B and L of Ramachandran plot is considered to be as accurate as a 2Å-resolution crystal structure [28]. Only 73.1% residues in the Geno3D generated model of BRBV 3D^pol^ are in the most favorable regions of Ramachandran plot (Figure 3A, Table 1A). In contrast SWISS-MODEL generated structure has 90.15% of its residues in the most favorable region of Ramachandran plot (Figure 3B, Table 1B). Other main chain parameters such as omega angle standard deviation, bad contacts/100 residues, zeta angle standard deviation, hydrogen bond energy standard deviation and overall G factor, which underscores to structural accuracy of a structure as determined by PROCHECK, also show that the SWISS-MODEL generated structure has better structural attributes (Tables 1A and 2A).

Side chain parameters were shown as chi1-chi2 side chain torsion angle combinations for all residue types whose side chains are long enough to have both these angles. The chi1-chi2 plots which were presented as side chain parameters in Tables 2A and 2B indicate SWISS-MODEL generated structure exhibited higher score in terms of overall structural accuracy. In conclusion, the results from PROCHECK analysis clearly suggested that the modeled structure of BRBV 3D^pol^ generated by SWISS-MODEL program was superior in quality. Therefore, further characterization of the modeled structure of BRBV was therefore, carried out on SWISS-MODEL generated structure.

### 2.5. Comparison of BRBV 3D^pol^ and Other 3D^pol^ Crystal Structures

**Figure 4:**
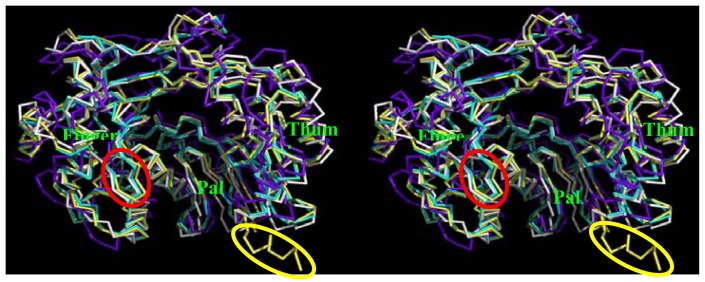
Stereoview of superimposed RdRp structures: A stereoview of ribbon representation of BRBV, HRV-16 and rabbit hemorrhagic disease virus (RHDV) RdRp 3D^pols^ superimposed on to foot and Mouth Disease virus (FMDV) 3D^pol^ (PDB, 1U09). Finger, palm and thumb domains are labeled. FMDV, BRBV, HRV-16 and RHDV 3D^pols^ are colored yellow, cyan, white and blue, respectively. An important loop, which aligns the template channel, is not present in RHDV 3D^pol^ (red oval). BRBV 3D^pol^ lacks the *C*-terminal α-helix in comparison to FMDV 3D^pol^ (yellow oval). The parts covering palm domain and the loop aligning T/P channel, have been removed to facilitate visualization of the regions with significant differences.

**Figure 5:**
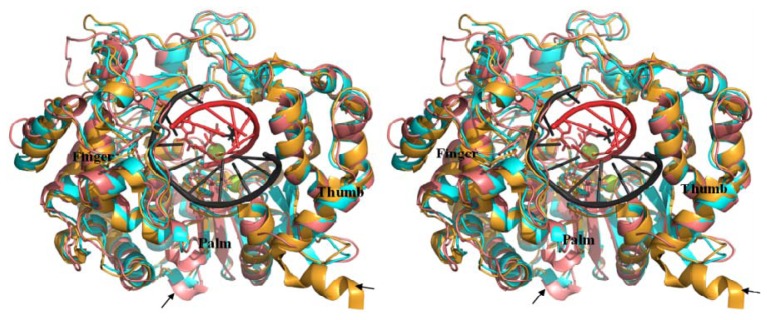
Stereoview of modeled BRBV 3D^pol^ superimposed on FMDV3D^pol^: Polymerases and RNA template/primer are presented as cartoon structures. Uridine 5′-triphosphate (UTP) and pyrophosphate (PPi) are presented as sticks. Metals (Mg^++^) are presented as green spheres. The modeled structures generated by Geno3D [A] and SWISS-MODEL [B] are colored pink and cyan, respectively. FMDV 3Dpol protein is colored as bright orange. The template and primer RNA are colored as gray and red, respectively. UTP and PPi are colored red, and gray, respectively. Finger, palm and thumb domains of the polymerase are marked. Major structural differences are highlighted with black arrows and discussed in the text.

PDB coordinate file can be found as supplementary information. As shown in Figure 4, the modeled structure of BRBV (cyan) and crystal structures of HRV-16 (white) as well as RHDV 3D^pol^ (blue) were superimposed on FMDV 3D^pol^ (yellow) (PDB, 1U09). BRBV Model showed high degree of homology to FMDV 3D^pol^ followed by HRV-16 and RHDV counterparts. The thumb domain of RHDV 3D^pol^ was observed to be shifted inwards as compared to the FMDV and other RdRps. RHDV polymerase lacks critical positively charged loop (shown in red oval, Figure 4), which aligns the template-binding surface observed in FMDV. BRBV 3D^pol^ appears to lack *C*-terminal α-helix as compared to FMDV counterpart (marked with yellow oval). FMDV 3D^pol^ *C*-α trace RMSD were 0.758, 1.518, and 4.607 for BRBV 3D^pol^ model, HRV-16 and RHDV 3D^pols^, respectively. It is important to note that the RMSD score of ≤1.0 Å indicates a very good agreement between the template and homology model. The comparison of three dimensional structures of the RdRps also supports the close relatedness of BRBV 3D^pol^ to its FMDV counterpart. As indicated with arrows in Figure 5 the modeled structure generated by Geno3D (pink color) has an important α-helix disordered in the finger domain. In addition, the loop aligning the template channel is pushed upwards in this model. In contrast the SWISS-MODEL generated structure (cyan color) has higher degree of conservation of these structural features in the context of FMDV 3D^pol^.

### 2.6. Analysis of Key Structural Features of FMDV 3Dpol and BRBV Homology Model

**Figure 6:**
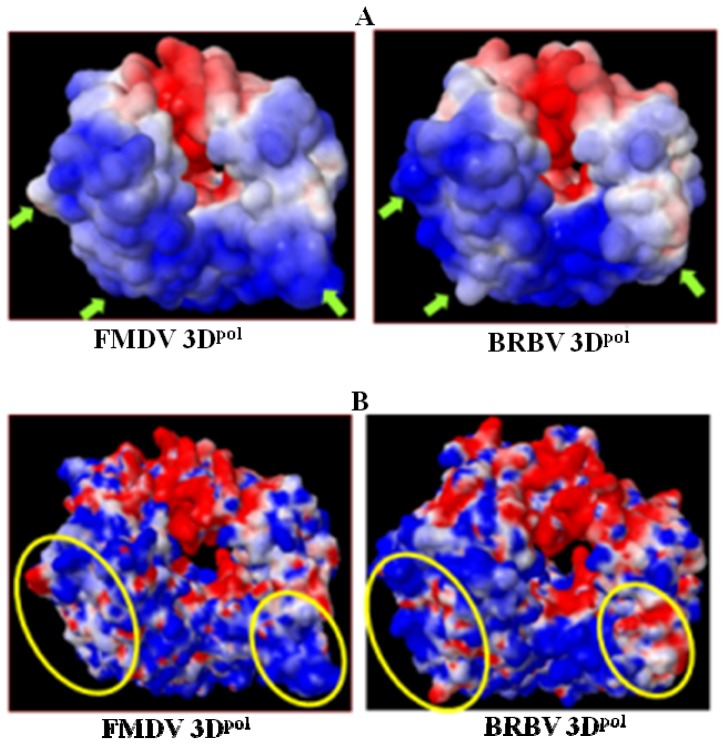
Electrostatic surface of BRBV 3D^pol^: Electrostatic surface of BRBV 3D^pol^ and FMDV 3D^pol^ are colored red to blue ranging from most negative (−5 kT/e) to most positively (+5 kT/e) charged regions. Neutral surface is shown in white color. Major differences in the distribution of solvent exposed and solvent excluded surface residues are indicated with green arrows (**A**) and yellow ovals (**B**).

The electrostatic surface potentials of modeled BRBV 3D^pol^ and FMDV 3D^pol^ apo enzyme were calculated using the Adaptive Poison Boltzman Solver (APBS) via PDB2PQR web server [29,30]. Solvent exposed and solvent excluded electrostatic surface structures were prepared for both FMDV and BRBV 3D^pol^ (Figure 6A,B). Total electrostatic energies were recorded to be in total 4.15 × 10^5^ and 4.29 × 10^5^ kilo-Joule per mole (kJ/mol), respectively for FMDV and BRBV 3D^pol^. The similar distribution of the electrostatic potential energy also substantiates similar structural attributes of the two polymerases. However, as reflected in Figure 6A,B the BRBV 3D^pol^ lacks the C-terminal α-helix with positively charged surface observed in FMDV counterpart. Other key differences in the distribution of positively charged, neutral and negatively charged residues have been indicated by green arrows and yellow ovals in Figure 6A,B, respectively. It is important to note that BRBV 3D^pol^ has more positively charged residues at the base of finger sub domain as opposed to FMDV 3D^pol^, which has more neutral amino acids in this region (white). In contrast the base of thumb sub domain is positively charged in FMDV 3D^pol^, C terminus of BRBV 3D^pol^ has an uncharged surface. These differences could individually or collectively contribute to physiochemical behavior as well as the function of the two enzymes.

### 2.7. Analysis on Non-Covalent Interactions in FMDV and BRBV 3D^pol^

Non-covalent interactions such as hydrogen bonds, salt bridges, van der waals interactions, hydrophobic interactions, cation-pi and CH-pi bonds are individually weak but collectively they determine the structure and behavior of proteins. Hydrogen bonds and salt bridges were calculated using VMD [31]. BRBV 3D^pol^ was observed to forms an extensive network of hydrogen bonds (46 hydrogen bonds) exactly similar to FMDV 3D^pol^. The distribution of these bonds was also very similar between FMDV and BRV 3D^pol^. Since hydrogen bonds are major contributors of protein folding and function, it is conceivable that given other similar features BRBV 3D^pol^ is homologous to its FMDV counterpart. However, the number of salt bridges was strikingly different between BRBV FMDV 3D^pol^, which form 1 and 17 salt bridges, respectively. Since salt bridges are critical for the thermal behavior of proteins it would be interesting to explore the temperature sensitivity of the protein and compare it to FMDV and other closely related proteins. The cation-pi interactions, formed between positively charged amino acids and aromatic amino acids, when they are in close proximity, contribute significantly to the protein structure [32]. Arginine (R) is more likely than lysine (K) to participate in a cation-pi interaction. The order of participation of aromatic side chains in cation-pi interactions is Tryptophan (W) > Tyrosine (Y)> Phenylalanine (F). We recorded 4 energetically significant cation-pi interactions in FMDV 3D^pol^, which exist between R17-F162, R153-W273, R388-W223, and K400-W462. In contrast BRBV 3Dpol possesses only 1 energetically favorable cation-pi interaction, which is formed between R385 and W220.

### 2.8. Analysis of Template/Primer (T/P) Binding Interface and Active Site of BRBV 3D^pol^

**Figure 7:**
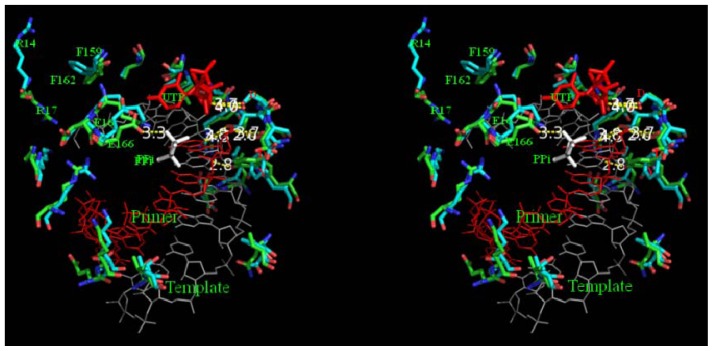
Stereoview of functionally important residues of BRBV 3D^pol^ and FMDV 3Dpol: Template/primer binding residues and active site of BRBV 3D^pol^ (cyan) and FMDV 3D^pol^ (green) are shown is stick representation. The nitrogen and oxygen atoms of the amino acids are colored blue and red, respectively. Template and primer are colored gray and red respectively and shown as lines. UTP and PPi are shown as sticks colored red and light gray, respectively. Amino acid residues are labeled as single letter amino acid codes with the identifiers. Catalytic tyrosine and glutamate residues are red colored one letter residue symbols. Arginine 14 and phenyl alanine 159 are flipped out of the nucleic acid binding interface in BRBV 3D^pol^. Hydrogen bonds are shown with yellow dotted lines and marked with bond length.

T/P binding interface of BRBV 3D^pol^ was mapped on to FMDV 3D^pol^ bound to decameric RNA, uridine tri-phosphate and pyrophosphate (PDB, 2E9Z). As shown in Figure 7, the template binding interface of both BRBV 3D^pol^ and FMDV 3D^pol^ are aligned with positively charged amino acids, which is an adaptation of the RdRp to accommodate negatively charged nucleic acid substrate. It is interesting to note that the side-chains of R14 and F 159 of BRBV have been flipped out of the plane in comparison to FMDV 3D^pol^. Their counterparts in FMDV 3D^pol^ form a strong cation-pi interaction. This might be a key stabilizing interaction, which holds the β-loop and keeps the T/P binding interface of the polymerase intact. The cation-pi interaction between W220 and R385 in BRBV 3D^pol^ could be an important determinant of its function. This bond is formed between β-13 strand and α-8 helix in the palm region that align the catalytic motif of the polymerase. Glutamate 163 is also moved out of the template channel losing an important hydrogen bond interaction with pyrophosphate in BRBV 3D^pol^ (Figure 7). Some of the differences in the bonding pattern could also be attributed to the use of apo structure of FMDV 3D^pol^ as a template for homology modeling. Since, it is an unliganded structure, there is more rotational freedom for the amino acids as compared to RNA and NTP bound polymerase. Such rotations may yield the altered conformations of amino acid side chains and hence alter the bonding pattern as well to some extent. Alternatively, it is also possible that in a replicating polymerase it is the alternate conformation of these amino acids that is predominant. Such structural rearrangements are gaining attention as a mechanism that RdRps adopt to facilitate the replication activity of their polymerases [10,11]. It is important to note that the available literature suggests that growth of BRBV in tissue culture is difficult to achieve [33] and virus infected cells incubated at 31 or 33 °C display more pronounced CPE than when incubated at 37 °C [2]. These studies also support a different physiological and thermal behavior of BRBV 3D^pol^ suggested herein on the basis of the structure and bonding pattern.

## 3. Experimental Section

### 3.1. Sequence

BRBV polypeptide sequence (NCBI Reference Sequence: YP_003355055.1) was utilized to retrieve the putative 3D^pol^ region, which was later utilized for sequence comparisons and homology modeling.

### 3.2. Phylogenetic Analysis

Phylogeny tree was prepared in order to determine the relatedness of BRBV 3D^pol^ to other related polymerases. The tree was calculated from distance matrices determined from percent identity or aggregate BLOSUM62 score using average distance as implemented in JalView [17,18]. In this method, pairwise distances used to cluster the sequences are represented as the percentage of mismatches between two sequences. The branch lengths are the percentage mismatch between two nodes; the leaves show the sequence IDs.

### 3.3. Structure based Alignment of the Polymerase Sequences

The structure based alignment of BRBV 3Dpol with other RdRps was performed using iterative magic fit function incorporated in the Deep view-[19]. The aligned sequences were manually curated to characterize the key structural motifs in each protein accurately. The key structural features such as functional motifs as well as T/P and nucleotide triphosphate interacting residues were assigned different color and symbols manually.

### 3.4. Preparation of Homology Model of BRBV 3D^pol^

The homology models of the protein were prepared via:

- Geno3D web server
  Crystal structures of FMDV (PDB, 1U09) and HRV-16 3D^pol^ (PDB, 1XR7) were utilized as templates for model generation. After the completion of run 10 homology models were prepared and their quality was assessed through PROCHECK [20,28]. Geno3D (http://geno3d-pbil.ibcp.fr) is an automated web server for protein molecular modeling [20]. Starting with a query protein sequence, the server performs the homology modeling in six successive steps: first, it identifies homologous proteins with known structures by using PSI-BLAST [21]. This provides the user all potential templates for target selection. After the user defines the templates and submits the job, the server performs the alignment of both query and subject sequences and extracts geometrical restraints (dihedral angles and distances) for corresponding atoms between the query and the template. Lastly, it performs the 3D construction of the protein by using a distance geometry approach.
- SWISS-MODEL
  The crystal structure of FMDV 3Dpol (PDB, 1U09) was identified as the closest match to the BRV counterpart and later utilized as template by SWISS-MODEL workspace, which is an integrated web-based modeling environment. For a given target protein it searches a library of experimental protein structures to identify suitable templates. On the basis of a sequence alignment between the target protein and the template structure, a three-dimensional model for the target protein is generated. The alignment produced by SWISS-MODEL was verified using another alignment algorithm (T-coffee). In homology modeling the most crucial steps is the evaluation and refinement of the raw model and SWISS-MODEL utilizes a set of unique analysis tools in addition to commonly tools such as PROCHECK and WHAT CHECK [22–26,28,34] to achieve this. These tools include atomic empirical mean force potential (ANOLEA) which performs energy calculations on a protein chain, evaluating the “Non- Local Environment” (NLE) of each heavy atom in the molecule and is used to assess packing quality of the models; QMEAN which is a composite scoring function for both the estimation of the global quality of the entire model as well as for the local per-residue analysis of different regions within a model; DFIRE, which is an all-atom statistical potential based on a distance-scaled finite ideal-gas reference state and reflects the quality of the model to indicate that a that a model is lower energy closer to the native conformation [35–37]. Finally, it minimizes the structure using GROMOS9639 performing 200 cycles by the steepest descent method and 300 cycles by the conjugate gradient method to minimize the steric clashes [38]. With stringent quality controls SWISS-MODEL workspace usually generates models with a reasonably reliable quality.

### 3.5. DeconSTRUCT Analysis of the Homology Models of BRBV 3D^pol^

In order to find the degree of homology of BRBV 3D^pol^ to other viral RdRps, the best scoring homology models of BRBV 3D^pol^ prepared by both SWISS-MODEL and Geno3D were submitted to the deconSTRUCT web server, which offers an interface to a protein database search engine that detects similar protein sub-structures. Firstly, it deconstructs the query structure into its SSEs and finds the match to the target by requiring a (tunable) degree of similarity in the direction and sequential order of SSEs. The search engine of deconSTRUCT utilizes the hierarchical organization and judicious use of the information about protein structures to achieve the sensitivity and specificity of the established search engines at orders of magnitude increased speed, without tying up irretrievably the substructure information in the form of a hash.

### 3.6. Calculation of Electrostatic Surface Potential

Electrostatic surface potential energies for BRBV and FMDV 3D^pol^ were calculated using APBS via PDB2PQR web portal (http://kryptonite.nbcr.net/pdb2pqr/) [29,30]. The input files were prepared from the PDB files of the two polymerases (BRBV and FMDV). Atomic charges and ionic radii were assigned according to Assisted Model Building with Energy Refinement **(**AMBER) force field. PROPKA was used to assign the protonation state to the proteins at pH 7.0. The proteins were assigned a low dielectric constant of 2.0 with a solvent dielectric constant of 78.54. Solvent accessible and solvent excluded surfaces of the two proteins were prepared. −5 and +5 kT/e (k is Boltzman Constant, T is temperature and e is the charge of electron) settings were used to color most positively charged and most negatively charged surface.

### 3.7. Calculation of Non-Covalent Interactions

The total number of hydrogen bonds was calculated in modeled as well as FMDV 3D^pol^ crystal structure, which was used as template to generate the model. The salt bridges were calculated using VMD [31]. Cation-pi interactions are formed when a cationic side-chain (Lys or Arg) is near an aromatic side-chain (Phe, Tyr, or Trp). Under such conditions, the geometry of aromatic ring is biased toward one that would experience a favorable cation-π interaction. Energetically favorable cation-pi interactions were alculated in both the modeled BRBV 3D^pol^ as well as FMDV 3D^pol^ structures via CaPTURE (http://capture.caltech.edu/) program developed by Justin Gallivan [32].

### 3.8. Preparation of Structures

Different representations of the structures of all the proteins were created Pymol version 1.3, wherever not mentioned otherwise [39].

## 4. Conclusions

In this study we built a high quality homology model of the 3D polymerase of bovine rhinitis B virus, which shares the highest homology to the FMDV counterpart. The modeled structures generated by different methods were characterized extensively for accuracy and relevance in terms of other closely related RdRps. We found that BRBV 3D^pol^ lacks *C*-terminal α-helix, which appears as an extension of thumb sub-domain in FMDV 3D^pol^. The active site and palm sub-domain show high conservation similar to most polymerases. The significant differences in the number and pattern of non-covalent interactions as compared to FMDV 3D^pol^ could be an important determinant of the thermal enzyme behavior of BRBV 3D^pol^. Although, the template binding (97.5%) and active sites (100%) are highly conserved between FMDV and BRBV 3D^pol^, the differences in adjacent residues, as well as bonding, could be important in determining the overall functionality of the protein. The finding of the present study would help broaden understanding of *Aphthovirus* RdRp, in particular, understanding the conformational changes in aphthoviral polymerases, which is as yet not an established phenomenon.

## Figures and Tables

**Table 1:** Alignment score from deconSTRUCT search.

PDB id	Aln. Score	Aln. Length	RMSD (Å)	Avg. dL	Geom. Z	Target Molecule
1U09A	330.50	370	1.42	1.88	−7.20	FMDV RdRp
2B43A	273.36	337	2.65	2.11	−4.04	Hepatitis C virus (HCV) RdRp
1KHWA	259.87	316	2.43	2.75	−3.44	RHDV RdRp
1S48A	182.59	231	2.87	2.50	0.51	Bovine viral diarrhea virus (BVDV Rna-dependent rna polymerase
2JL9A	116.57	148	2.94	1.67	1.58	Phi6 RNA polymerase

Aln. Score is the alignment value obtained from the web server after the search for the closely resembling protein structures available in PDB; Aln. Length is the length of the residues aligned to the query sequence.

**Table 2A:** Summary of main chain parameters for the structure generated by Geno3D.

Stereochemical quality	No. of data points	Parameter value	Comparison typical value	Value band width	No. of band widths from mean	Interpretation
% residues in A,B,L	413	73.6	83.8	10	−1.0	WORSE
Omega angle St. Dev.	463	0.6	6.0	3.0	−1.8	BETTER
Bad Contact/100 Residue	0	0	4.2	10	−0.4	INSIDE
Zeta angle St. Dev.	433	1.1	3.1	1.6	−1.3	BETTER
H-bond Energy St.Dev.	283	0.7	0.8	0.2	−0.5	INSIDE
Overall G-Factor	465	0.2	−0.4	0.3	1.9	BETTER

St. Dev. denotes the standard deviation of the score observed. The accuracy of structure is depicted in the order BETTER > INSIDE > WORSE each parameter.

**Table 2B:** Summary of main chain parameters for the structure generated by SWISS-MODEL.

Stereochemical quality	No of data points	Parameter value	Comparison typical value	Value band width	No. of band widths from mean	Interpretation
% residues in A,B,L	405	90.1	76.6	10	1.4	WORSE
Omega angle St. dev.	452	5.2	6.0	3.0	−0.3	BETTER
Bad Contact/100 Residue	0	0	10.5	10	−1.1	INSIDE
Zeta angle St. Dev.	425	1.0	3.1	1.6	−1.3	BETTER
H-bond Energy St. Dev.	320	0.7	0.9	0.2	−1.1	INSIDE
Overall G-Factor	454	0.1	−0.6	0.3	2.2	BETTER

St. Dev. denotes the standard deviation of the score observed. The accuracy of structure is depicted in the order BETTER > INSIDE > WORSE each parameter.

**Table 3A:** Summary of side chain parameters for the structure generated by Geno3D.

Stereochemical quality	No. of data points	Parameter value	Comparison typical value	Value band width	No. of band widths from mean	Interpretation
Chi-1 gauche minus St. Dev.	52	10.0	22.7	6.5	−1.9	BETTER
Chi-1 trans St. Dev	118	10.1	22.7	5.3	−2.4	BETTER
Chi-1 gauche plus St. Dev.	200	10.1	21.3	4.9	−2.3	BETTER
Chi-1 pooled St. Dev.	370	10.2	22.0	4.8	−2.4	BETTER
Chi-2 transst dev	108	8.6	23.1	5.0	−2.9	BETTER

St Dev. denotes the standard deviation of the score observed. The accuracy of structure is depicted in the order BETTER > INSIDE > WORSE each parameter.

**Table 3B:** Summary of side chain parameters for the structure generated by SWISS-MODEL.

Stereochemical quality	No. of data points	Parameter value	Comparison typical value	Value band width	No. of band widths from mean	Interpretation
Chi-1 gauche minus St. Dev.	60	16.6	18.1	6.5	−0.2	INSIDE
Chi-1 trans St. Dev.	132	11.9	19	5.3	−1.3	BETTER
Chi-1 gauche plus St. Dev.	185	13.8	17.5	4.9	−0.8	INSIDE
Chi-1 pooled St. Dev.	377	14.2	18.2	4.8	−0.8	INSIDE
Chi-2 trans St. Dev.	86	14.7	20.4	5.0	−1.1	INSIDE

St. Dev. denotes the standard deviation of the score observed. The accuracy of structure is depicted in the order BETTER > INSIDE > WORSE each parameter.
